# Effect of NaCl Partial Replacement by Chloride Salts on Physicochemical Characteristics, Volatile Compounds and Sensorial Properties of Dry-Cured Deer Cecina

**DOI:** 10.3390/foods10030669

**Published:** 2021-03-21

**Authors:** Marcio Vargas-Ramella, José M. Lorenzo, Rubén Domínguez, Mirian Pateiro, Paulo E. S. Munekata, Paulo C. B. Campagnol, Daniel Franco

**Affiliations:** 1 Centro de Educação Superior da Região Sul—CERES da Universidade do Estado de Santa Catarina, Laguna 88790-000, Brazil; marcio.ramella@hotmail.com; 2 Centro Tecnológico de la Carne de Galicia, Rúa Galicia N° 4, Parque Tecnológico de Galicia, San Cibrao das Viñas, 32900 Ourense, Spain; jmlorenzo@ceteca.net (J.M.L.); rubendominguez@ceteca.net (R.D.); mirianpateiro@ceteca.net (M.P.); paulosichetti@ceteca.net (P.E.S.M.); 3 Área de Tecnología de los Alimentos, Facultad de Ciencias de Ourense, Universidad de Vigo, 32004 Ourense, Spain; 4 Departmento de Tecnologia e Ciência de Alimentos, Universidade Federal de Santa Maria, Santa Maria 97105-900, Brazil; paulocampagnol@gmail.com

**Keywords:** deer meat, free fatty acids, free amino acids, lipid oxidation, volatile compounds, dry-cured meat, sensory analysis

## Abstract

The present study aimed to evaluate the effect of NaCl replacement in the physicochemical quality and volatile and sensorial profile of dry-cured deer cecina. Two salt mixtures were used as NaCl substitute: mixture I (30% NaCl-70% KCl) and mixture II (30% NaCl-50% KCl-15% CaCl_2_-5% MgCl_2_). Regarding the physicochemical parameters, only ash content, pH and L* values were affected by NaCl replacement. However, lipid oxidation was affected by NaCl replacement. The greatest thiobarbituric acid reactive substances (TBARS) values were observed in the control batch (3.28 mg MDA/kg). The partial replacement of NaCl by salt mixtures affected (*p* < 0.001) Ca, K, Mg, and Na content. The total amounts of free fatty acids and free amino acids were not affected (*p* > 0.05) by NaCl replacement. Concerning the volatile compounds, control samples presented the highest concentrations of furans (*p* < 0.01), while samples produced with mixture II had the lowest (*p* < 0.001) amounts of esters and acids. Our results indicated that all sensory attributes of the attribute map were affected (generalized procrustes analysis (GPA) explained 100% of the total variability among treatments). Considering the results obtained from the sensorial analysis, only mixture II reduced the overall acceptance and preference of consumers. Control attained significantly (*p* < 0.05) greater scores of acceptance and preference than mixture II despite the higher TBARS content.

## 1. Introduction

Cecina is a dry-cured meat (salted, smoked and dried) that can be elaborated from several species, different pieces of meat, and variations in the processing conditions [1]. This product is appreciated in several Mediterranean countries [2], similar products can be found in many other areas of the world [1]. Sodium chloride (NaCl) is the most important component in the cecina elaborating process, because of its key role in meat quality parameters such as water-holding capacity and water activity. Moreover, it has a central role in proteolysis and lipolysis phenomena and, therefore, in product shelf-life [3,4]. Proteolysis and lipolysis events in meat are the most important mechanisms with consequences in sensory quality (such as texture, aroma, and flavour) of cecina [5]. Although lipid oxidation generally has negative effects on meat and meat products, in some cases it contributes to the development of pleasant aromas. In fact, it is well known that the compounds derived from lipid oxidation play an important role in the development of the typical aroma of meat products during ripening or dry-cured stages, which is one of the most appreciated attributes by consumers.

Overall, the large increase in the consumption of salted foods worldwide has resulted in a general NaCl intake that has exceeded recommended dietary values (from ~9 to 12 g/day) [4,6]. In this context, the World Health Organisation (WHO) has agreed to reduce the global population’s intake of salt by 30% by 2025 [6]. Therefore, following scientific information and health recommendations, meat companies are trying to develop low-salt products to reduce dietary sodium consumption. Decreasing and replacing NaCl intake has been identified as one of the most cost-effective measures to improve the population’s health, especially with potassium chloride [6]. Likewise, utilization of calcium (CaCl_2_) and magnesium (MgCl_2_) salts has been successfully employed in meat salted products as a sodium substitute [7].

Although there have been advances in the development of salt replacing ingredients for dry-cured meat, there is still a negative impact associated with these substitutes in several characteristics (especially in texture, flavour, appearance, moisture, and shelf-life) of meat products [4,8]. Despite the cecina processing has been previously studied for several species such as equine [5,9,10,11,12], ovine [1], bovine [3,13,14], deer [14], and caprine [14], the effects of NaCl replacement by other chloride salts in this product was assessed only with equine meat [11]. Due to the technological importance of NaCl in dry-cured meat products be comprised of many aspects, the effect of salt reduction appears to be highly product-specific [4].

Additionally, an increasing number of consumers are demanding meat with high nutritional value (rich in protein, and low in lipid and cholesterol amount), tasty and obtained from lower intensive systems using natural resources [15]. In general, deer meat meets with these requirements, presenting low fat and cholesterol content and a high proportion of essential amino acids and long-chain polyunsaturated fatty acids [16,17]. In this sense, fresh meat from deer could be considered as one of the red meat alternatives to beef. Moreover, the replacement of NaCl by other chloride salts in the processing of dry-cured deer cecina could provide an important opportunity to enhance perception of deer meat and meat products as “healthy food” among consumers. Previous studies have reported the use of deer meat in the production of meat products such as loin [18], patê [19], burger [20], and sausage [21] but, to the best of our knowledge, there are no studies that assess meat quality characteristics of dry-cured deer cecina. With the premises that cecina will presumably present good overall acceptability by consumers [2] due to high nutritional value and sensorial properties of deer meat [16,17], it is expected that deer cecina can fulfil a market requirement.

Therefore, considering the challenge of reducing the sodium content without significant reduction in the quality of dry cured meat products, this study aimed to assess the partial substitution of NaCl with KCl, CaCl_2_, and MgCl_2_ salts on the physicochemical parameters, volatile compounds profile, and sensorial characteristics of dry-cured deer cecina for the first time.

## 2. Materials and Methods

### 2.1. Cecina Manufacture

Pieces of meat were obtained from animals (males and females) with similar age (from 30 and 40 months) and carcass weight (±35 kg). No significant differences were detected for sex effect in meat chemical composition (unpublished data). Cecina production was repeated twice in two different months, but the raw material was obtained from the same hunting season within 30 days to reduce the season variability effect in results. Forty-eight knuckles (quadriceps femoris composed of vastus lateralis, vastus intermedius, vastus medialis, and rectus femoris muscles) of 1.42 ± 0.22 kg from hunted red deer were employed. Raw pieces were randomly divided into three groups and salted by immersing them in a saturated brine (9 kg of salt mixtures per 30 L of water). Cecinas from the control batch were salted with NaCl (100% NaCl), whereas the salt mixture I was composed of 30% NaCl and 70% KCl and the salt mixture II of 30% NaCl, 50% KCl, 15% CaCl_2_ and 5% MgCl_2_. All cecinas were salted for 2 h/kg at 2–5 °C with a relative humidity (RH) range between 85% and 90%. After the salting stage, pieces were washed and transferred to a post-salting chamber (35 days at 2–5 °C and 85–90% RH). Then, cecinas were smoked using oak wood in a cooking-smoking chamber (Jugema, mod. KWE-1, Industrial Fluerpla S.L., Valencia, Spain) at 25 °C during 2 h and then they were placed in a dry chamber at 12–14 °C and 75–78% RH for 15 days and at 15–17 °C and 65–70% RH for additional 45 days. At the end of the process, all cecinas were analysed. The production of cecinas was replicated using the same ingredients and protocols in two different months. The cecina samples to conduct the analysis were obtained from three central slices of each cecina (cut from the widest part of the cecina), with approximately 2 cm in length per slice. After the pH and colour assessment and the separation of the samples for texture test, slices were minced and homogenized for the remaining analyses.

### 2.2. Proximate Composition Analysis

The moisture [22], protein [23], and ash [24] contents were determined following the international standards organization (ISO) procedures, while total fat was measured according to American Oil Chemistry Society official method [25].

### 2.3. Physicochemical Analysis

The pH values were obtained using a portable pH-meter (Hanna Instruments, Eibar, Spain) equipped with a penetration probe. A portable colorimeter (Konica Minolta CM-600d, Osaka, Japan) with the following components was employed to determinate colour parameters (L*—brightness, a*—greenness/redness and b*—blueness/yellowness) in the CIELAB space: pulsed xenon arc lamp, angle of 0° viewing angle geometry, standard illuminant D65 and aperture size of 8 mm [26]. For texture analysis [Warner–Bratzler test (WB) and texture profile analysis test (TPA)] a texturometer (TA.XT.plus of Stable Micro Systems, Vienna Court, UK) was utilized. To carry out the WB shear force test, three cooked meat pieces of 1 cm × 1 cm × 2.5 cm (height × width × length) were removed from cecinas (cuts were made parallel to the muscle fibre direction).

Samples were completely cut using a shear blade with a triangular slot cutting edge (1 mm of thickness) and maximum shear force performed to cut the sample was obtained. Force-time curves were recorded at a crosshead speed of 3.33 mm/s and recording speed was also 3.33 mm/s. Lipid oxidation was measured by thiobarbituric acid reactive substances (TBARS) index (2-thiobarbituric acid; secondary products of the lipid oxidation). Physicochemical analysis were assessed following the procedures described by Barros et al. [27].

### 2.4. Free Fatty Acids Analysis

Twenty mg of extracted fat were separated through an NH_2_-aminopropyl solid phase extraction (SPE) column [28], and transesterified with sodium methoxide and sulfuric acid–methanol solutions following the method described by Barros et al. [27]. The free fatty acids were identified and quantified using a gas chromatograph (GC-Agilent 7890B, Agilent Technologies, Santa Clara, CA, USA) with a flame ionization detector (FID), following the chromatographic conditions reported previously by Barros et al. [27]. Results were expressed as g/100 g of fat.

### 2.5. Mineral Analysis

The mineral elements (Ca, Fe, K, Mg, Mn, Na and P) were quantified by inductively coupled plasma-optical emission spectroscopy (ICP-OES) using a Thermo-Fisher ICAP 6000 plasma emission spectrometer (Thermo-Fisher, Cambridge, UK). The mineral results were expressed as mg/100 g (except for manganese, which was expressed as µg/100 g)

### 2.6. Free Amino Acid Analysis

Free amino acids were extracted following the method proposed by Lorenzo et al. [29] and analysed by reversed-phase high performance liquid chromatography (RP-HPLC) using a Waters 2695 Separations Module with a Waters 2475 Multi Fluorescence Detector, equipped with a Waters AccQ-Tag amino acids analysis column. The derivatization process as well as the chromatographic conditions were indicated previously in another study [30]. Results were expressed as mg/100 g of dry matter.

### 2.7. Volatile Compounds Analysis

The extraction, separation and identification of volatile compounds of deer cecinas were analysed using a solid phase microextraction (SPME)-gas chromatography–mass spectrometry 7890B GC-System (Agilent Technologies, Santa Clara, CA, USA) equipped with a mass selective detector 5977B MSD (Agilent Technologies), following the procedure described by Domínguez et al. [31]. Lineal retention index (LRI) was calculated for DB-624 capillary column (30 m × 0.25 mm id, 1.4 μm film thickness; J&W Scientific, Folsom, CA, USA) and installed on a gas chromatograph equipped with a mass selective detector. The volatile results were expressed as area units per gram of sample (AU × 10^4^/g of sample).

### 2.8. Sensory Analysis

The generalized procrustes analysis (GPA) was conducted with 12 trained panellists selected from the Meat Technology Centre of Galicia. The cecinas were individually coded with a three-digit number, cut into slices of approximately 5 mm thick and served at room temperature on white plastic dishes. Slices were individually labelled with three-digit random numbers. Eleven sensory attributes of deer cecinas, grouped according to appearance (red colour of lean, marbled, and yellow and pink colour of fat), flavour, odour, saltiness and texture (hardness, juiciness, fibrousness and pastiness) were evaluated following ISO regulations [32,33,34]. The intensity of every attribute was rated in a structured scale from 0 (sensation not perceived) to 10 (maximum of the sensation) and the results were expressed as the mean scores of each attribute given by all the panellists.

Sensory acceptability analysis was conducted by 36 consumers (aged between 29 and 40 years and from both genders) from Ourense (Spain) in three sessions. The treatments were evaluated to determine whether the consumer liked or disliked the deer cecinas salted with different salt mixtures in comparison to control. To avoid the possible order of presentation effect, slices were presented to consumers in a random order [35] and served with water and toast. Consumers evaluated the deer cecinas by the acceptance test using a 7-point hedonic scale, which ranged from “1—disliked much” to “7—liked much”. Additionally, consumers were asked to order the samples according to their preference, using a structured 3-point scale (1 = most favourite and 3 = less favourite). The study was approved by the local committee of Centro Tecnológico de la Carne(SEN/2020).

### 2.9. Statistical Analysis

Forty-eight cecinas were used in the present study (8 cecinas × 3 batches × 2 manufacture process replicates). After checking the normal distribution and variance homogeneity (Shapiro–Wilk test), data from proximate composition, physicochemical parameters, free fatty acids, free amino acids, minerals and volatile compounds were examined using a one-way analysis of variance (ANOVA). Duncan’s test was used for the determination of differences between least squares means (*p* < 0.05). All statistical analysis were carried out with the IBM SPSS Statistics program for Windows (version 25.0. IBM Corp., New York, NY, USA).

The XLSTAT-Sensory version 2018 (Addinsoft SARL, Paris, France) software was employed to evaluate all sensory data. A GPA was used to minimize the differences between panellists. Configurations of three cecinas samples, by eleven sensory parameters, and by 12 panellists, were combined to find consensus. The GPA was applied for the significantly different attributes and it was conducted to evaluate the deer cecinas and, in this way, create the attribute maps. For quantitative descriptive analysis (QDA), a two-way mixed model ANOVA was conducted, with panellist and treatment as independent variables. The dependent variables were the intensity ratings corresponding to each sensory attribute. Tukey’s mean separation tests were chosen for post hoc analyses (*p* < 0.05). The Friedman two-way ANOVA, assuming product and taster as an independent factor, was used to analyse the obtained data of preference test. When a significant effect (*p* < 0.05) was found, the least significant difference (LSD) was used as a multiple comparison test. 

## 3. Results and Discussion

### 3.1. Chemical Composition and Physicochemical Parameters of Dry-Cured Deer Cecina

In Table 1 is shown the effect of NaCl replacement on the proximate composition and physicochemical parameters of cecina. The partial replacement of NaCl by salt mixtures significantly affected (*p* < 0.05) ash, pH, L*, and TBARS values. Cecina prepared with salt mixture I (30% NaCl and 70% KCl) showed the greatest (*p* < 0.05) values for ash, pH, and L* (mixture I > mixture II > control), while control group presented higher (*p* < 0.05) values for TBARS assay than both alternative treatments.

Our results partially disagree with those observed in a previous study with foal cecina [11] prepared with similar salt mixtures (100% NaCl vs. 50% NaCl and 50% KCl vs. 45% NaCl, 25% KCl, 20% CaCl_2_ and 10% MgCl_2_). In that study, the authors found that NaCl partial replacement affected (*p* < 0.05) pH, proximate composition (moisture, protein, and ash), and colour (L*, a*, and b*). By contrast, in the present study the ash content was significantly (*p* < 0.001) higher in cecina with salt substitutes (mixture I > mixture II > control). The mineral content at the end of the processing reflects the salt penetration and diffusion through the cecina whole piece [11,36]. However, our results seem to be inconsistent with another study [37] that found lower ash content in batches containing CaCl_2_ and MgCl_2_. Unexpected, it seems that the presence of divalent cations in the present study did not slow down salt penetration into the inner portions of the piece when compared to control (100% NaCl). A possible explanation could be the fact that, despite divalent cations penetrating less than monovalent cations in the muscle, CaCl_2_ and MgCl_2_ have a greater water solubility compared to NaCl [37].

The pH value was significantly (*p* < 0.001) higher in cecinas elaborated with the mixture I (6.17) than control (5.94) and mixture II (5.83) treatments. Our pH results agree with those published by Vidal et al. [7] who found that meat salted with CaCl_2_ had lower (*p* < 0.05) pH value than salted with NaCl and KCl. The reduction in pH of meat products produced with di-cationic salts was previously reported by several studies [38,39,40]. The decrease in pH values with the addition of CaCl_2_ is associated with the two atoms of Cl^−^, leading to lower pH values when compared to the monovalent salts NaCl and KCl [7]. During the ripening stage, the colour of fresh meat changes from brilliant red to dark red, which is characterized by a decrease in the luminosity (L*) [10]. According to our assays, higher (*p* < 0.05) values for L* (30.71) in cecinas were observed in treatment with larger KCl percentage (mixture I). In other words, the cecinas produced with mixture I were brighter than the other two treatments. The range of L* values obtained in the present study (between 25.85 and 30.71) agree with intervals reported in the literature (between 25.10 and 32.30) for cecina obtained from several species [3,5,9,10,11,12,13].

The TBARS values differed significantly (*p* < 0.05) between salt mixtures treatments and control. The higher malonaldehyde content obtained in the control batch could be related to the NaCl percentages. As previously reported by Andrés et al. [41], higher NaCl content in brines tends to increase TBARS values. In addition, the pro-oxidative effects of NaCl during dry-cured meat processing could reduce or inhibit the activity of antioxidant enzymes (e.g., superoxide dismutase) and promote lipoxygenase (the main enzyme that initiates lipid oxidation) [42,43]. This result may be explained by the capacity of NaCl to disrupt the cellular membrane integrity, which leads to the access of oxidant agents to lipid substrates and consequently to the liberation of ions from iron-containing molecules [42].

### 3.2. Mineral Composition of Dry-Cured Deer Cecina

The influence of different salt mixtures in the mineral composition of cecinas is shown in Table 2. The partial replacement of NaCl by salt mixtures affected (*p* < 0.001) Ca, K, Mg, and Na content. As expected, the mineral composition of the cecinas agreed with the formulation of each treatment, hence significant differences (*p* < 0.001) were detected for Ca, K, Mg and Na.

The WHO recommends that adults (≥16 years of age) consume less than 2 g/day of sodium (equivalent to 5 g salt/day) and >3.5 g/day of potassium to contribute to healthy blood pressure, avoiding risks associated with heart diseases and strokes [6]. Sodium content obtained for control (~1.844 g/100 g of cecina) is close to the maximum sodium intake recommended meanwhile alternative treatments are close to the recommended intake for potassium (3.17 and 2.46 g for treatments I and II respectively). From a nutritional point of view, the use of mixtures rich in potassium displayed that not only can they significantly (*p* < 0.001) reduce the sodium content (in less than half) of the cecina but they can also improve (*p* < 0.001) its potassium amount. Additionally, potassium is a basic nutrient for the maintenance of the total body in terms of acid and electrolyte balance, as well as the regular cell function [6]. Moreover, the use of CaCl_2_ and MgCl_2_ has been proposed in food formulations as an alternative since these salts in dry-cured meat products could provide an important supplementation to prevent some diseases such as osteoporosis and hypertension [4,7,8,36,37,44,45].

### 3.3. Free Fatty Acids Content of Dry-Cured Deer Cecina

Free fatty acid (FFA) profile was determined as an indicator of the lipolysis process. In Table 3 is shown the effect of NaCl replacement on the FFAs amount (expressed as g/100 g of fat) of cecina. For the profile quantification, 41 fatty acids were identified but only those that represented ≥0.1% were included in the table (19 fatty acids). Nevertheless, all fatty acids were considered for calculating saturated fatty acid (SFA), monounsaturated fatty acid (MUFA), polyunsaturated fatty acid (PUFA), n-3, n-6, and total FFAs.

Proteolysis and lipolysis play a key role in the development of dry-cured meats flavour [46]. In the present study, no significant (*p* > 0.05) effect of chloride salts on total contents of SFAs, MUFAs, PUFAs, n-3, n-6, and total FFAs was found. Similar outcomes were obtained by Cittadini et al. [11] evaluating NaCl replacement in dry-cured foal cecinas, with no significant differences in percentage of SFA, MUFA, or PUFA between batches. In our product, the FFAs predominantly determined were pentadecanoic (C15:1n-5), linoleic (C18:2n-6), palmitic (C16:0), stearic (C18:0), and oleic fatty acids (C18:1n-9). However, only pentadecanoic acid (C15:0) presented significant (*p* < 0.01) differences among batches (control > mixture I and mixture II; 0.185 > 0.151 and 0.137). The profiles of the most important fatty acids obtained were similar to those previously published by Lorenzo et al. [5] for foal cecina (oleic > palmitic > linoleic > stearic > linolenic).

As previously reported by Armenteros et al. [47], in the present study higher concentrations of divalent cations in salt formulations (mixture I and mixture II) were not capable of decreasing the lipolysis process by reducing the accumulation of FFA. Our results indicate that the lipolysis phenomenon in alternative treatments (mixture I and II) occurred in a very similar way to that with 100% NaCl. These findings agree with those previously presented [11,36] in cecina salted with different chloride salts (Na, KCl, Ca_2_, and Mg_2_) were none of the replacements affected (*p* > 0.05) total FFAs content. There is a correlation between length of dry-cured meat processing, especially during ripening stage, and FFA content [48]. Due to the activity of phospholipases in phospholipids and lipases in triacylglycerols in the muscle, the fatty acids are gradually released during the processing of cecina [10]. In the present research, FFAs represented 35.1–38.52% of the total fat. Our values were greater than those found for foal cecina (12.71–26.50%) [11,12,49] and dry-cured lacón (14.62–22.52%) [29] with similar length of the drying-curing process (~80 days). 

### 3.4. Free Amino Acids Content of Dry-Cured Deer Cecina

In Table 4 is shown the effect of NaCl replacement on the free amino acids (FAA) profile (expressed as mg/100 g of dry matter) of cecina.

For total FAA, no significant (*p* > 0.05) effect of NaCl replacement was found. Results were in disagreement with those published previously by Cittadini et al. [11] in foal cecinas in which the authors reported significant (*p* < 0.001) variations in total contents of FAA among salt-curing formulations (100% NaCl vs. alternative chloride salts). In our study, the main FAA observed were leucine, phenylalanine, and alanine for all batches. Furthermore, the FAA with significant (*p* < 0.05) differences among treatments were leucine, phenylalanine, alanine, valine, isoleucine, methionine, cysteine, and proline. Except for proline and valine, higher (*p* < 0.05) values of these amino acids were displayed in cecinas salted with alternative salt mixtures than in control samples. It is also relevant to mention that partial replacement of sodium has been related to significantly greater contents of FAAs in dry-cured meat products [29,36].

Several factors can be attributed to discrepancies in the FAA profile observed among the aforementioned studies. The enzymatic activities promoted by aminopeptidases, catepsins, and exopeptidases are directly involved in the FAA releasing in dry-cured meat. Aminopeptidases are the main enzymes causing the hydrolysis of amino acids from peptides and proteins, and could be active during the entire drying-curing process [36,50,51]. Nonetheless, several factors could modify their activity, which would affect amino acid release [50]. The increment in FAA content in dry-cured meat products manufactured with reduced-sodium salt mixtures could be attributed to the activity of these enzymes. Indeed, several authors have previously reported a higher aminopeptidase (leucil, arginil and alanyl) [36], cathepsin D and exopeptidases [51] activities in brines with KCl, Ca, and Mg. Moreover, previous studies [5,11,29,52,53] indicated higher proteolysis in the final product when sodium was replaced in the formulation of curing salt. Additionally, according to Armenteros et al. [52] divalent salts (CaCl_2_ and MgCl_2_) and KCl could have lower inhibitory effects on muscle enzyme activity than NaCl if used in different concentrations.

Significant differences (*p* < 0.05) observed in the FAA profile between batches of dry-cured deer cecina appears to indicate that chloride salts act as a modulating agent of the enzymatic activities during the processing. This fact could be probably due to the effect of salting treatment in the degradation of polypeptides to FAA mainly by the action of proteolytic enzymes [36]. Moreover, some of these free amino acids are of great importance because they may be involved in volatile compounds generation, contributing to flavour development in the dry-cured product [36,51]. Therefore, it is very important to establish adequate NaCl replacement because it may have important consequences on enzyme activity, and consequently, on sensorial properties.

### 3.5. Volatile Compounds of Dry-Cured Deer Cecina

A total of 125 volatile compounds were identified in the final products. These compounds were classified into 10 chemical families: alcohols, hydrocarbons (Table 5), ketones, sulphur compounds, esters (Table 6), aldehydes, furans, acids, phenol and benzene derived compounds and others volatile compounds (Table 7). A significant difference among batches was observed in esters (*p* < 0.001), furans (*p* < 0.01), acids (*p* < 0.001), and other compounds (*p* < 0.001) as well as in total volatile compounds (*p* < 0.001). The substitution of NaCl by mixture I (30% NaCl and 70% KCl) significantly decreased (*p* < 0.01) furans and increased (*p* < 0.001) others volatile compounds (Table 7). Additionally, the use of mixture II (30% NaCl, 50% KCl, 15% CaCl_2_, and 5% MgCl_2_) produced a decrease (*p* < 0.01) in esters (Table 6), furans, acids and total volatile compounds (Table 7). From our data, it seems that the presence of CaCl_2_ and MgCl_2_ of salt mixture II could decrease the formation of total volatile compounds in the cecina. The effect of NaCl reduction has shown controversial findings in previous studies [54,55] that assessed the volatile compounds profile. These studies demonstrated that strategies to reduce NaCl influence the formation of volatile compounds. In addition, our hypothesis disagrees with previous authors [56] who found that salting formulation with 55% NaCl, 25% KCl, 15% CaCl_2_, and 5% MgCl_2_ led to a more intense production and release of the most abundant chemical families of volatiles.

**Figure 1:**
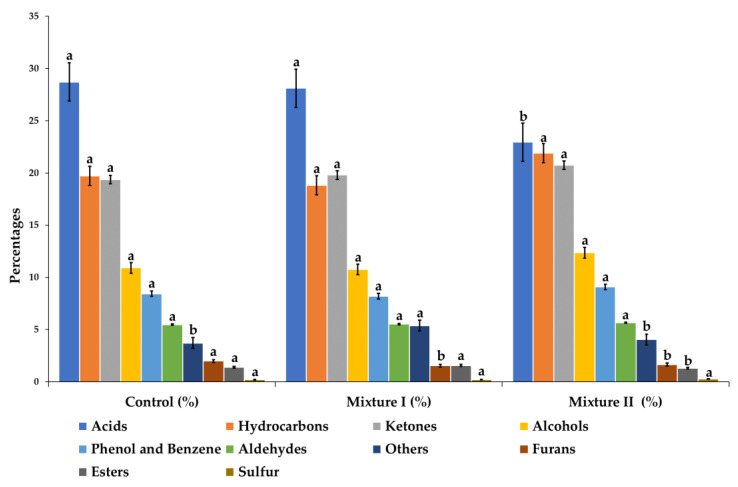
Chemical families of volatile compounds (percentages respect to total chromatographic area) according to the three treatments (Control: 100% NaCl; Salt mixture I: 30% NaCl and 70% KCl; salt mixture II: 30% NaCl, 50% KCl, 15% CaCl_2_, and 5% Mg_2_). ^a,b^ Mean values± standard error (corresponding to the same parameter) followed by a different letter differ significantly (*p* < 0.05; Duncan’s test).

Considering the total chromatographic area, the most abundant chemical families of volatile compounds were acids, hydrocarbons, and ketones (Figure 1). Concerning the control and mixture II treatments, the most abundant families were the acids (28.72% and 22.95%, respectively), followed by hydrocarbons (19.73% and 21.90%, respectively) and ketones (19.37% and 20.74%, respectively). On the contrary, in the salt mixture I, the most prominent groups were acids (28.12%), ketones (19.80%), and hydrocarbons (18.83%).

Our outcomes do not support previous research with venison cecina [14] in which samples showed hydrocarbons as the most abundant group. On the contrary, another study [12] working with cecina reported esters as the major group, followed by hydrocarbons and aldehydes. A study [56] evaluating NaCl replacement by KCl, CaCl_2_, and MgCl_2_ with different formulations in dry-cured hams found the following volatile compound relationship among chemical families: aldehydes > hydrocarbons > acids > alcohols > ketones > esters. According to these authors, the generation of volatiles was drastically affected by the salt formulations and significant (*p* < 0.001) differences were observed in the total amount of volatile compounds.

To understand these findings, it is important to consider the influence of processing conditions, seasonings, and biochemical and chemical reactions that take place during the processing of cecinas [10]. Amino acid degradation, carbohydrate fermentation, lipid oxidation, and microbial esterification are the complex and main processes involved in the formation of volatile compounds in cecina [10,14]. In addition, the incorporation of smoke-derived compounds into meat pieces is another important factor in the production of cecina, which have undergone an intense smoking process in the present study. It should be noted that the intensity of smoking process after the post-salting stage is dependent of the manufacturer preferences [14]. Additionally, although modifications in the process could affect volatile profiles it should be noted that only a few volatile compounds contribute to the aroma and they must be at a concentration above their olfactory threshold [55].

Acids reached the highest concentrations of total chromatographic area in the three studied treatments (22.95–28.72%). Otherwise, this class of compounds has a moderate impact in flavour due to their relatively intermediate odour threshold values (0.05–9 ppm) [55]. The presence of acids could be a result of the smoking stage in which species and condiments are added [57]. The production of acids is also a consequence of amino acid catabolism caused by microbial fermentation of *staphylococci* present in meat products [55]. Moreover, this catabolism during microbial activity is a precursor in the formation of ester compounds that gradually accumulate during processing [12,57]. However, cecina is a dry-cured meat characterized by a short process (~90 days). Hence, this period of processing was short to observe the degradation of accumulated acids into esters.

Our results for hydrocarbons agree with a previous research [12] with foal cecina by indicating this family as the second most abundant classes of volatile compounds. However, these compounds have a reduced impact on the flavour of dry-cured meats [10,14]. Another main group of volatile compounds in all studied groups (in a similar proportion to hydrocarbons) was the ketones. This result agrees with another study for venison cecina [14] that indicated ketones as one of the predominant class of volatile compounds. Ketones can be produced via lipid autoxidation and/or microbiological metabolism of dominant microbial species in dry-cured meat [14,43]. Nonetheless, our findings suggest that lipid oxidation did not affect final ketone composition. The results of lipid oxidation assay showed high (*p* < 0.05) values of TBARS for control batch, but no statistical differences (*p* > 0.05) were observed among treatments for this family of volatiles. Similarly, our data also could indicate that microbiological pathway as total ketones source was not affected by salt replacement treatments. Despite ketones having high odour threshold values in comparison to other compounds [55], they are considered to have a huge importance in meat products’ aromas. High concentrations of these compounds impart peculiar odour that can be perceived as ethereal, butter, spicy, and blue cheese notes [12].

Regarding alcoholic volatile compounds concentration, our results disagree with another study with dry-cured ham [56] which reported that alcohols were affected (*p* < 0.001) by the salting formulations applied. The authors [56] indicated percentages of total alcohols considerably higher in dry-cured hams elaborated with 55% NaCl, 25% KCl, 15% CaCl_2_, and 5% MgCl_2_. Despite alcohols being mainly generated as reaction products from lipid oxidation [57], the authors [56] suggested that this fact was probably due to a more intense chemical reduction of the corresponding branched aldehydes by microbial enzymes. In our study, no significant (*p* > 0.05) differences were observed among batches. Once the sensory attributes are affected by volatile compounds [58], it should be noted that alcohols have a low odour threshold and contribute to meat flavour (e.g., herbaceous, woody, and fatty notes) [57].

Phenol and benzene-derived families were the fifth most abundant group (8.20–10.09%) in deer cecina. Results were lower than those found by a previous study [57] that indicated phenols as the second most predominant group of compounds in smoked dry-cured hams (23.98%). Phenolic compounds (phenols and metoxyphenols) are mainly responsible for the unique aroma and taste of smoked products. Phenols have low threshold value so their effect in the flavour of smoked meat is considerable [14,57]. Apart from initial raw material used for cecina elaboration, the variations obtained in phenol content are explained by differences in the smoking phase as well as the type of wood used in the process [57].

Regarding aldehydes, our results (~5.56%) disagree with previous studies that found this family as a relevant group in the volatile composition of dry-cured loin (~20.5%) [49] and the major group in dry-cured hams [56,57]. Analysing the effect of salt replacement, Purriños et al. [59] found significant differences (*p* < 0.001) for the sum of all aldehydes among salting treatments in dry-cured lacón. Among the volatile compounds derived from lipid oxidation, aldehydes are one of the most abundant [60]. Aldehydes have a low perception threshold which highlight their importance in the aroma of final products even at trace amounts [14,51]. Indeed, they are probably the most interesting lipid-derived volatile compounds because of their wide range of flavours and odours [60]. The low concentration of aldehydes evaluated in the present study could be the reflection of the mechanism of formation of these compounds or their participation in other chemical reactions. Aldehydes are derived from lipid oxidation [mainly from the linoleic (C18:2n−6) and arachidonic acids (C20:4n−6)] [10] and the bacterial metabolism of amino acids (mainly phenylalanine and leucine) [55].

Concerning the group “others”, the highest (*p* < 0.001) levels were identified in cecinas salted with the mixture I (I: 30% NaCl and 70% KCl). According to Hierro et al. [14], this group is predominantly composed of volatile compounds typically found in wood smoke. In this group, amines, amino acids, hydrogen sulfide, thiols, and ammonia led to the generation of different heterocyclic compounds (e.g., pyrroles, pyrazines). These volatile compounds have a low odour threshold and may contribute to nutty and roasted notes [55].

Furan volatile compounds were higher in the control group (100% NaCl). Furans were previously described because of salting [12] and smoking stage (characteristic compounds of wood smoke) [14] in dry-cured meat, meanwhile, they were not detected in raw pieces [12].

Regarding esters, they were identified in low amounts (~1.43% of the total chromatographic area). Our results disagree with those previously published for dry-cured foal [12,49] in which esters were reported as the most abundant group. Ester volatile compounds are formed by esterification of carboxylic acids and alcohols by microorganisms that increment with longer ripening stages [57]. Cecina is dry-cured meat that is characterized by having a short curing period (60–120 days) [10]. Therefore, considering the points already discussed for acids, the cecina processing length could also affect the formation of esters. Finally, sulphur compounds were the volatiles that presented the lowest percentage (~0.23%). These volatile compounds are characterized by a very low odour threshold and formed by proteolysis of sulphur amino acids (cysteine and methionine) [49,55].

The effect of salt replacement on each family of volatile compounds within batches is discussed below. The volatile compounds were identified and classified in the following chemical families: alcohols, and hydrocarbons (Table 5); ketones, sulfur compounds, and esters (Table 6); and aldehydes, furans, acids, phenol and benzene derived compounds, and others (Table 7).

#### 3.5.1. Alcohols, and Hydrocarbons

In Table 5 is shown the effect of NaCl replacement on the alcohols and hydrocarbons content (expressed as area units per gram of sample, AU × 10^4^/g of cecina). 

The most abundant alcohols were furan-3-methanol and propane-1,2-diol. Seven alcohol volatile compounds presented significant (*p* < 0.05) differences among treatments, five of them with higher values in cecinas elaborated with alternative salts. The salt mixture I presented the highest values for 3-methylbutan-1-ol and phenylmethanol, while salt mixture II showed the greatest values for prop-2-en-1-ol and hexan-1-ol. Previous studies [56] have found considerably higher (*p* < 0.001) amounts of some alcohols compounds in batches when KCl, CaCl_2_, and MgCl_2_ were present. This fact could be attributed to the well-known NaCl antimicrobial activity, hence the partial replacement of NaCl can result in a more intense microbial growth and a greater production of alcohols from microbial spoilage [56]. Additionally, it has been demonstrated previously that salt reduction and substitution with KCl provoked the formation of some derived alcohols [55]. On the other hand, most abundant hydrocarbons were decane; 3-ethylpentane; 2,3,3-trimethylpentane; 2,2-dimethylpropane; and tridecane. Previous research has demonstrated that this group is not involved significantly in dry-cured meat overall flavour because they have high flavour thresholds [51].

#### 3.5.2. Ketones, Sulphur Compounds, and Esters

In Table 6 is shown the effect of NaCl replacement on the ketones, sulfur compounds, and esters content (expressed as AU × 10^4^/g of cecina).

Twenty-five ketones were identified in cecina, being the 3-hydroxybutan-2-one and 1-hydroxypropan-2-one the most abundant compounds of this chemical family in all treatments. However, only 3-hydroxybutan-2-one and propan-2-one presented significant (*p* < 0.05) differences among treatments. 3-hydroxybutan-2-one was greater (*p* < 0.05) in cecinas salted with mixture I when compared to mixture II. This ketone has a central role in the final aroma by imparting butter and cheese odour to cecina. As stated previously, dominant microbial species can form ketones (butan-2-one and 3-hydroxybutan-2-one) in the final product [14]. Thus, unlikely previously discussed for total ketones, it seems that salt formulation affected the metabolism of microorganisms that produces specific compounds such as 3-hydroxybutan-2-one.

The most abundant esters were ethenyl acetate, 3-methylbutyl acetate and 3-methylbutyl acetate. Around 59% of total esters were represented by ethenyl acetate. Our results were different than those published for dry-cured foal loin [49]. In this case, the most abundant esters (around 79% of the total) were propyl 3-methylbutanoate, methyl propanoate and methyl acetate. Esters can modify the global flavour due to their low odour threshold values, imparting fruity notes and ripened flavour in cured meat products [14,48]. Moreover, these compounds positively affect the flavour as a typical attribute of aged meat [60]. The presence of some ethyl esters (such as methyl acetate and methyl propanoate) could be explained by the esterification activity of staphylococci and lactic acid bacteria among other microorganisms in meat products [61].

#### 3.5.3. Aldehydes, Furans, Acids, Phenol, Benzene-Derived Compounds, and “Others”

In Table 7 is shown the effect of NaCl replacement on the aldehyde, furans, acids, phenol, benzene-derived, and “others” content (expressed as AU × 104/g of cecina).

Among the aldehydes, 2-phenylacetaldehyde was the most abundant. This molecule is related to acorn, rancid and pungent sensations [57]. Our results disagree with previous studies that found hexanal as the principal component of aldehydes in dry-cured meat products such as cecinas [12,14] and hams [56,57]. In the present study, hexanal was the fourth most abundant aldehyde. This volatile has been described as the greatest indicator of lipid oxidation in dry-cured meat [51,56,60,62]. High amounts of hexanal indicates flavour deterioration, often resulting in an aroma described as rancid [54,57,60], strong, hot, green leaves, vegetables [14], and grass [57]. Our results disagree with those published previously in the dry-cured hams [56]. According to the authors, significantly higher hexanal content was obtained from products using 100% NaCl and another formulation with 50% NaCl and 50% KCl than in dry-cured hams salted with 55% NaCl, 25% KCl, 15% CaCl_2_, and 5% MgCl_2_. Even though we found higher (*p* < 0.05) TBARS values in the control treatment than in alternative salt groups, the control and treatment II presented higher (*p* < 0.01) values for hexanal than the treatment I. Our results also disagree with those published previously about lipid oxidation promoted by KCl in Jinhua dry-cured ham [54]. In that study, the authors found that replacing NaCl by KCl had a pro-oxidative effect and resulted in increased lipid oxidation and the formation of hexanal.

The 2-pentylfuran was the most predominant furan. This compound is a result of the salting stage and is a noncarboxylic compound with a relatively low threshold that contributes to a vegetable aromatic note (green fruit) [58]. Derived from linoleic acid and other n-6 fatty acids, 2-pentylfuran has been described as the most abundant furan in previous studies with salted meat [54,58] as well as, in dry-cured meat products manufactured from whole pieces, such as cecina, loin and ham [12]. Its presence could play an important role in the overall flavour, along with as an indicator of lipid oxidation [57].

Considering volatiles compounds from acids group, our results were very similar to previous studies performed [10,49] to evaluate dry-cured meats. The authors of these studies reported 3-methylbutanoic acid; propanoic acid, and acetic acid as the most abundant compounds. These carboxylic acids might have a positive influence on the aroma due to conversion into fruity esters [56].

Finally, in the group “others”, 2,3,5-trimethylpyrazine; 2,6-dimethylpyrazine; 2,3-dimethylpyrazine; and pyrazine showed significant (*p* < 0.01) differences. Three of these compounds (2,3,5-trimethylpyrazine; 2,6-dimethylpyrazine; and 2,3-dimethylpyrazine) were significantly (*p* < 0.001) higher in samples salted with the mixture I (30% NaCl and 70% KCl) than in other treatments. The “others” group of compounds are typical from smoked products, as mentioned before [14]. As already discussed for ashes in proximate composition, the mineral content in the final product reflects its diffusion through the cecina. Therefore, it seems that higher content of volatile compounds from others group in the salt mixture I may be explained by the presence of potassium in this formulation. Potassium chloride was previously reported as a salt that easily penetrates the muscle due to its higher water solubility [37]. For this reason, brine with 70% of KCl could have contributed to the diffusion of these components in cecina.

### 3.6. Sensorial Analysis of Dry-Cured Deer Cecina

**Figure 2:**
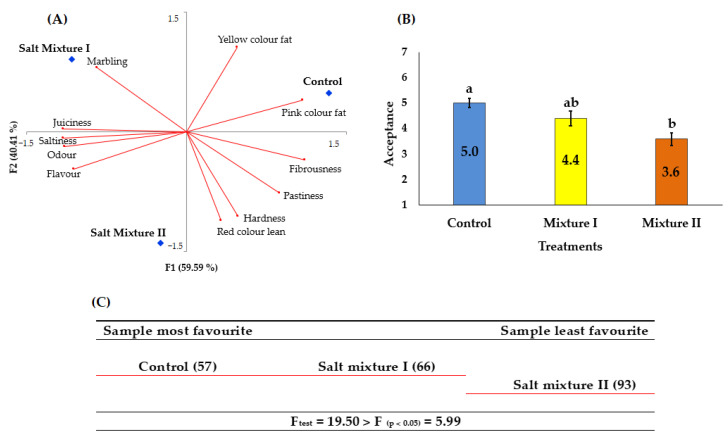
Sensorial profile (**A**), Overall acceptance (**B**), and Preference values and least significant difference (LSD) (**C**) of dry-cured deer cecina elaborated with three different treatments (Control: 100% NaCl; Salt mixture I: 30% NaCl and 70% KCl; Salt mixture II: 30% NaCl, 50% KCl, 15% CaCl_2_ and 5% MgCl_2_). ^a,b^ Mean values± standard error (corresponding to the same parameter) followed by a different letter differ significantly (*p* < 0.05; Tukey’s test). Preference test and LSD results: samples in the same row does not differ significantly (*p* > 0.05) and samples in different rows differ significantly (*p* ≤ 0.05). Numbers in brackets are Σ score.

During the processing of cecina, intensive biochemical reactions occur and lead to noticeable changes in its composition. The NaCl replacement by chloride salts (e.g.,: KCl and CaCl_2_) in dry-cured meat processing is well known to have a significant effect on meat composition and, consequently, in the sensorial properties of the final product [4,8]. For instance, the use of KCl in large amounts can cause negative impacts in meat products from a sensory point of view (metallic and bitter flavour) [7,56], meanwhile, CaCl_2_ can promote undesirable effects on flavour (bitter, metallic and residual taste), texture, and release of water in salted meat [7]. In the present study, the sensory properties of dry-cured deer cecina were influenced (*p* < 0.05) by the salt formulation. In Figure 2 is shown the sensorial profile (A), overall acceptance (B), and preference values and LSD (C) from dry-cured deer cecina. F1 and F2 dimensions obtained from the GPA explained 100% of the total variability among treatments (59.59% and 40.41% for F1 and F2, respectively). According to Rodrigues and Teixeira [63], the GPA test is used to minimize the differences between panel assessors, identify the consensus between them, and summarize results, thereby making it easier to interpret and identify the main conclusions.

The attributes more affected by F1 were yellow colour of fat, hardness and red colour of lean, whereas the pink colour of fat, fibrousness, pastiness, marbling, juiciness, saltiness, odour, and flavour had a higher impact in F2 (Figure 2A). As can be observed, cecina was characterized by an intense pink colour of fat in the control, marbling in treatment I, and red colour of lean and hardness in treatment II. Saltiness, juiciness, and pastiness were very low in all the samples studied. Considering F1 and the attributes related with this axis, the spatial separation indicated that control was explained in terms of the positively correlated attributes (yellow colour of fat) while treatment II was separated based in attributes negatively correlated with this axis (hardness and lean colour). Additionally, concerning second axis (F2), control was explained in terms of the positively correlated attributes (pink colour fat) while treatment I was explained in terms of the negatively correlated attributes (marbling, juiciness, saltiness, odour and flavour).

Regarding the acceptance test (Figure 2B), despite control and mixture I cecinas showed differences in acceptance according to the tasting panel (scores 5.0 and 4.4 corresponds to “like slightly” and “neither like nor dislike” respectively), no significant (*p* > 0.05) variation was observed between them. Our results for score acceptance were lower than those previously published for dry-cured ewe cecina with overall acceptability values in the range 6.41–6.58 [1] and 6.00–6.83 [2].

The preference values obtained with the order test [64] showed that from 36 consumers, 21 (58% of the total) chose the control as the preferred cecina and 15 (42% of the total) chose treatment I cecina. Cecina from treatment II was not chosen by any consumer as preferred. Finally, total scores of preferences (number in brackets in Figure 2C) showed that cecinas from the control and mixture I were preferred among the consumers. Friedman’s test indicated that there were significant differences (*p* < 0.05) among treatments (F_test_ > F_0.05_). The results of the LSD test (Figure 2C) showed that the cecinas can be clustered in two well-differentiated groups: one group composed of control and treatment I, and another group composed of treatment II. Considering the results obtained from the sensorial analysis, salt substitution by mixture II compared to the control batch modify the global acceptance and preference of consumers. Control attained significantly (*p* < 0.05) higher scores for acceptance and preference than formulation II despite the higher TBARS content. Divalent cations could produce metallic, astringent, and irritative sensations [56]. This fact might explain the low acceptability and preference of cecina produced with formulation II.

## 4. Conclusions

Our findings showed that the physicochemical composition, volatile compounds, and sensory characteristics of dry-cured deer cecina were affected by salting treatments. From a nutritional point of view, no significant effect of treatments was observed in proximate composition (moisture, fat and protein) and in total amounts of SFAs, MUFAs, and PUFAs. Despite instrumental analysis not showing significant differences among treatments that could negatively affect texture, colour, aroma, and flavour, cecinas differed most regarding the sensory analysis. A taste panel differentiated batches and indicated that mixture II modified the overall acceptance of deer cecina. The sensorial analysis showed that cecina manufactured with 100% NaCl and salt mixture I had the highest acceptance scores. In addition, according to a consumer preference test, no significant difference was observed between the control and treatment I. Thus, salt reformulation strategies in the development of dry-cured meat products are likely to have important impacts on sensory characteristics.

## Figures and Tables

**Table 1:** Effect of NaCl replacement by other chloride salts on chemical composition and physicochemical parameters of dry-cured deer ‘‘cecina’’.

	Control	Salt Mixture I	Salt Mixture II	SEM	Sig.
Proximate composition (g/100 g)					
Moisture	31.98	30.22	31.60	0.52	ns
Fat	1.45	2.39	2.19	0.18	ns
Protein	56.32	55.99	55.18	0.45	ns
Ash	8.01 ^c^	9.71 ^a^	8.71 ^b^	0.15	***
pH	5.94 ^b^	6.17 ^a^	5.83 ^c^	0.030	***
Colour parameters					
L*	25.85 ^b^	30.71 ^a^	26.09 ^b^	0.81	*
a*	3.44	3.33	3.19	0.16	ns
b*	3.19	4.61	3.11	0.34	ns
TBARS (mg MDA/kg)	3.28 ^a^	2.60 ^b^	2.41 ^b^	0.14	*
Texture parameters					
Firmness (N/s)	12.57	10.76	8.19	0.82	ns
Total work (N mm)	311.84	326.59	268.11	19.14	ns
Shear force (N/cm^2^)	43.046	37.29	31.08	2.33	ns

Control: 100% NaCl; Salt mixture I: 30% NaCl and 70% KCl; Salt mixture II: 30% NaCl, 50% KCl, 15% CaCl_2_ and 5% MgCl_2_; MDA = malondialdehyde Sig.: significance; ns: not significant; * *p* < 0.05; *** *p* < 0.001; ^a–c^ Mean values in the same row (corresponding to the same parameter) followed by a different letter differ significantly (*p* < 0.05; Duncan’s test).

**Table 2:** Effect of NaCl replacement by other chloride salts on the mineral content of dry-cured deer ‘‘cecina’’.

Minerals (mg/100 g)	Control	Salt Mixture I	Salt Mixture II	SEM	Sig.
Ca	13.55 ^b^	12.08 ^b^	265.85 ^a^	18.62	***
Fe	7.95	7.64	7.06	0.24	ns
K	851.66 ^c^	3169.45 ^a^	2459.89 ^b^	163.01	***
Mg	46.21 ^b^	44.10 ^b^	104.09 ^a^	4.48	***
Mn (µg/100 g)	39.42	42.56	46.34	2.09	ns
Na	1844.86 ^a^	759.20 ^b^	706.73 ^b^	82.30	***
P	476.25	462.54	471.66	7.21	ns

Control: 100% NaCl; Salt mixture I: 30% NaCl and 70% KCl; Salt mixture II: 30% NaCl, 50% KCl, 15% CaCl_2_ and 5% MgCl_2_; Sig.: significance; ns: not significant; *** *p* < 0.001; ^a–c^ Mean values in the same row (corresponding to the same parameter) followed by a different letter differ significantly (*p* < 0.05; Duncan’s test).

**Table 3:** Effect of NaCl replacement by other chloride salts on free fatty acids of dry-cured deer ‘‘cecina’’.

Free Fatty Acids (g/100 g of fat)	Control	Salt Mixture I	Salt Mixture II	SEM	Sig.
C14:0	0.42	0.40	0.36	0.023	ns
C14:1n-5	0.12	0.13	0.11	0.0096	ns
C15:0	0.19 ^a^	0.15 ^b^	0.14 ^b^	0.0061	**
C15:1n-5	6.21	5.60	5.60	0.16	ns
C16:0	6.01	5.41	5.41	0.16	ns
C16:1n-7	0.79	0.79	0.74	0.047	ns
C17:0	0.31	0.25	0.26	0.011	ns
C17:1n-7	0.081	0.071	0.075	0.0036	ns
C18:0	5.98	5.23	5.46	0.16	ns
11t-C18:1	0.30	0.32	0.26	0.014	ns
C18:1n-9	4.73	4.58	4.92	0.22	ns
C18:1n-7	0.70	0.74	0.71	0.032	ns
C18:2n-6	6.06	5.41	5.61	0.18	ns
C18:3n-3	1.60	1.34	1.29	0.068	ns
C20:3n-6	0.25	0.23	0.26	0.012	ns
C20:4n-6	2.64	2.47	2.75	0.11	ns
C20:5n-3	0.65	0.59	0.57	0.027	ns
C22:5n-3	0.91	0.87	0.92	0.036	ns
C22:6n-3	0.19	0.18	0.18	0.011	ns
SFA	13.05	11.61	11.78	0.32	ns
MUFA	12.67	11.94	12.21	0.41	ns
PUFA	12.47	11.26	11.75	0.38	ns
n-6	9.07	8.22	8.76	0.28	ns
n-3	3.35	2.98	2.95	0.13	ns
Total free fatty acids	38.52	35.15	36.04	0.91	ns

Control: 100% NaCl; Salt mixture I: 30% NaCl and 70% KCl; Salt mixture II: 30% NaCl, 50% KCl, 15% CaCl_2_ and 5% MgCl_2_; saturated fatty acid (SFA), monounsaturated fatty acid (MUFA), polyunsaturated fatty acid (PUFA) Sig.: significance; ns: not significant; ** *p* <0.01; ^a,b^ Mean values in the same row (corresponding to the same parameter) followed by a different letter differ significantly (*p* < 0.05; Duncan’s test).

**Table 4:** Effect of NaCl replacement by other chloride salts on free amino acids of dry-cured deer ‘‘cecina’’.

Free Amino Acids (mg/100 g Dry Matter)	Control	Salt Mixture I	Salt Mixture II	SEM	Sig.
Aspartic acid	6.01	4.62	5.40	0.32	ns
Serine	66.78	77.21	73.91	3.39	ns
Glutamic acid	72.06	89.31	93.58	5.19	ns
Glycine	47.46	58.73	47.99	2.46	ns
Histidine	57.65	69.77	74.37	3.19	ns
Taurine	114.34	117.04	128.12	6.58	ns
Arginine	85.45	95.51	100.69	4.27	ns
Threonine	99.25	117.94	114.23	4.76	ns
Alanine	151.09 ^b^	206.50 ^a^	166.96 ^b^	7.66	**
Proline	59.83 ^a^	70.06 ^a^	44.45 ^b^	3.17	**
Cysteine	34.99 ^b^	44.05 ^b^	60.23 ^a^	3.49	**
Tyrosine	85.71	98.26	103.75	3.75	ns
Valine	132.20 ^b^	170.32 ^a^	157.17 ^a,b^	6.15	*
Methionine	79.47 ^b^	102.12 ^a^	102.56 ^a^	3.82	*
Lysine	81.62	103.37	104.76	5.71	ns
Isoleucine	117.78 ^b^	148.45 ^a^	147.91 ^a^	5.69	*
Leucine	249.77 ^b^	315.94 ^a^	330.03 ^a^	12.26	*
Phenylalanine	152.09 ^b^	187.83 ^a^	183.80 ^a^	6.55	*
Total free amino acids	1690.36	2074.54	2034.20	77.56	ns

Control: 100% NaCl; Salt mixture I: 30% NaCl and 70% KCl; Salt mixture II: 30% NaCl, 50% KCl, 15% CaCl_2_ and 5% MgCl_2_; Sig.: significance; ns: not significant; * *p* < 0.05; ** *p* < 0.01; ^a,b^ Mean values in the same row (corresponding to the same parameter) followed by a different letter differ significantly (*p* < 0.05; Duncan’s test).

**Table 5:** Effect of NaCl replacement by other chloride salts on alcohols, and hydrocarbons of dry-cured deer ‘‘cecina’’.

Volatile (AU × 10^4^/g of Cecina)	*m*/*z*	LRI	Control	Salt Mixture I	Salt Mixture II	SEM	Sig.
Cyclobutanol	44	489	4.90	3.71	4.08	0.25	ns
Methanethiol	48	492	1.37 ^a,b^	1.27 ^b^	1.73 ^a^	0.076	*
Propan-1-ol	59	563	21.23	23.57	17.64	1.01	ns
2-methylpropan-1-ol	43	643	5.90	5.19	6.69	0.31	ns
3-methylbutan-1-ol	70	817	133.38 ^b^	168.32 ^a^	120.68 ^b^	7.22	*
2-methylbutan-1-ol	57	821	36.36	33.30	29.34	1.69	ns
Pentan-1-ol	70	859	20.35	16.81	20.39	1.01	ns
Propane-1,2-diol	45	887	172.17	172.08	155.07	10.05	ns
Hexane-1,6-diol	59	910	5.37	7.22	5.77	0.38	ns
Prop-2-en-1-ol	57	933	79.92 ^b^	88.46 ^b^	130.14 ^a^	6.71	**
Hexan-1-ol	56	975	37.20 ^b^	22.30 ^c^	46.61 ^a^	2.27	***
Furan-3-ylmethanol	98	986	326.71	304.58	312.89	10.28	ns
Butane-1,2-diol	59	995	22.78 ^b^	32.44 ^a^	26.29 ^a,b^	1.57	*
3-ethyl-4-methylpentan-1-ol	69	1001	14.04 ^a^	17.28 ^a^	9.86 ^b^	0.85	***
Oct-1-en-3-ol	57	1079	151.20	126.29	119.37	7.08	ns
3-methylsulfanylpropan-1-ol	106	1106	10.82	11.46	7.46	0.82	ns
Phenylmethanol	108	1159	81.48 ^b^	94.45 ^a^	79.46 ^b^	2.64	*
4-methylphenol	107	1218	41.53	42.15	37.13	1.60	ns
2-phenylethanol	91	1221	139.83	150.49	109.50	9.02	ns
Undecan-1-ol	97	1299	3.03	3.49	3.15	0.21	ns
2-methoxy-4-prop-2-enylphenol	164	1394	3.33	4.09	2.99	0.28	ns
Total alcohols			1312.99	1328.95	1246.23	26.32	ns
Pentane	43	500	8.98 ^a,b^	7.65 ^b^	10.36 ^a^	0.45	*
3-methylpentane	56	542	1.24	1.29	1.34	0.066	ns
Hexane	57	600	134.71	117.53	115.89	4.50	ns
2-methylhexane	85	620	0.76 ^c^	1.22 ^b^	1.72 ^a^	0.083	***
3-methylhexane	71	633	0.71	0.89	0.86	0.045	ns
Heptane	71	700	23.76 ^b^	33.41 ^a^	36.33 ^a^	1.04	***
3-ethylpentane	71	759	422.57 ^a^	254.19 ^b^	232.08 ^b^	32.68	*
2,3,3-trimethylpentane	70	767	395.27 ^b^	437.89 ^a^	454.87 ^a^	6.37	***
2,3-dimethylhexane	70	775	43.98 ^b^	101.96 ^a^	108.49 ^a^	6.04	***
(E)-3,4-dimethylhex-2-ene	83	779	35.79 ^c^	41.96 ^b^	46.82 ^a^	0.96	***
2-methylheptane	57	782	4.43	3.51	4.15	0.22	ns
Octane	85	800	40.47	39.49	39.90	2.08	ns
2,3-dimethylheptane	84	906	44.24	46.68	49.30	0.97	ns
3,4-dimethylheptane	70	909	14.54	15.37	15.41	0.50	ns
2,2-dimethylpropane	57	927	247.01	240.39	204.98	11.90	ns
2,6,6-trimethylbicyclo[3.1.1]hept-2-ene	93	1000	21.85 ^a^	15.83 ^b^	22.74 ^a^	0.96	**
Decane	57	1000	758.56	769.19	658.41	47.48	ns
Tridecane	71	1300	153.55 ^b^	177.51 ^a,b^	193.22 ^a^	6.41	*
Tetradecane	57	1400	17.09 ^a^	14.44 ^a^	6.48 ^b^	0.89	***
Pentadecane	71	1500	2.63	2.36	2.49	0.13	ns
Lineal hydrocarbons			1139.79	1161.59	1063.09	49.89	ns
Branched hydrocarbons			1232.39	1161.18	1142.76	29.37	ns
Total hydrocarbons			2372.14	2322.77	2205.84	66.73	ns

Control: 100% NaCl; Salt mixture I: 30% NaCl and 70% KCl; Salt mixture II: 30% NaCl, 50% KCl, 15% CaCl_2_ and 5% MgCl_2_; Volatile results are expressed as area units per gram of sample (AU × 10^4^/g of sample). Sig.: significance; ns: not significant; * *p* < 0.05; ** *p* < 0.01; *** *p* < 0.001; ^a–c^ Mean values in the same row (corresponding to the same parameter) followed by a different letter differ significantly (*p* < 0.05; Duncan’s test). *m*/*z*: Quantifier ion.

**Table 6:** Effect of NaCl replacement by other chloride salts on ketones, sulfur compounds, and esters content of dry-cured deer ‘‘cecina’’.

Volatile (AU × 10^4^/g of Cecina)	*m*/*z*	LRI	Control	Salt Mixture I	Salt Mixture II	SEM	Sig.
Propan-2-one	58	518	22.47 ^b^	40.45 ^a^	17.44 ^b^	1.87	***
Butan-2-one	72	586	20.99	25.25	20.90	1.28	ns
Pentan-2-one	86	722	2.39	2.79	2.75	0.17	ns
1-hydroxypropan-2-one	43	726	402.56	317.67	426.42	23.03	ns
Pentane-2,3-dione	100	738	3.93	5.25	4.07	0.31	ns
3-hydroxybutan-2-one	45	795	1001.61 ^a,b^	1217.43 ^a^	835.24 ^b^	54.50	*
4-methylpentan-2-one	100	803	1.65	1.66	1.43	0.0934	ns
1-hydroxybutan-2-one	57	873	156.20	129.66	133.20	8.99	ns
Cyclopentanone	84	883	21.93	23.14	24.47	0.84	ns
Cyclohexanone	98	940	13.28	13.84	13.03	0.67	ns
Cyclopent-2-en-1-one	82	947	18.86	20.60	18.67	0.94	ns
3-methylcyclopentan-1-one	98	952	5.68	6.48	4.71	0.36	ns
2-methylcyclopent-2-en-1-one	96	1017	107.45	116.27	105.72	5.52	ns
1-(furan-2-yl)ethanone	110	1026	85.81	86.07	72.84	4.21	ns
Oxolan-2-one	86	1072	51.96	42.73	45.35	2.05	ns
3-methylcyclopent-2-en-1-one	96	1095	109.65	109.99	95.31	4.59	ns
1-(furan-2-yl)propan-2-one	95	1117	17.07	16.15	15.27	0.83	ns
4-methyl-2H-furan-5-one	98	1126	75.07	67.83	67.24	2.83	ns
2-hydroxy-3-methylcyclopent-2-en-1-one	112	1144	124.93	111.59	106.67	6.54	ns
2-hydroxy-3,4-dimethylcyclopent-2-en-1-one	126	1164	10.05	9.40	9.18	0.44	ns
3,4,5-trimethylcyclopent-2-en-1-one	124	1167	11.88	12.24	11.06	0.64	ns
Phenacyl formate	105	1172	11.37	11.81	10.83	0.56	ns
1-cyclopropylpropan-1-one	69	1206	43.35	44.37	39.63	2.33	ns
1-(3,5-dihydroxyphenyl) ethanone	137	1331	6.34	7.75	5.87	0.42	ns
2,3-dihydroinden-1-one	104	1354	2.31	2.34	2.19	0.10	ns
Total ketones			2328.79	2442.78	2089.49	66.54	ns
Methylsulfanylmethane	62	520	4.91 ^b^	6.39 ^a^	3.45 ^c^	0.27	***
Methanedithione	76	524	19.73 ^b^	21.23 ^a,b^	24.19 ^a^	0.76	*
Total sulphur compounds			24.64	27.63	27.64	0.80	ns
Methyl acetate	74	529	3.03 ^b^	3.27 ^b^	4.34 ^a^	0.19	**
Ethenyl acetate	86	581	109.68 ^a^	121.71 ^a^	65.68 ^b^	6.34	***
Ethyl propanoate	57	740	6.51 ^b^	10.89 ^a^	6.76 ^b^	0.48	***
3-methylbutyl acetate	88	930	28.76	31.26	24.53	1.77	ns
3-methylbutyl acetate	70	959	13.63 ^b^	18.12 ^a,b^	19.28 ^a^	0.99	*
2-methylbutyl acetate	70	962	2.07	3.51	3.62	0.29	ns
Propyl 3-methylbutanoate	85	1032	5.03	5.99	6.60	0.31	ns
Total esters			168.72 ^a^	194.75 ^a^	130.82 ^b^	6.74	***

Control: 100% NaCl; Salt mixture I: 30% NaCl and 70% KCl; Salt mixture II: 30% NaCl, 50% KCl, 15% CaCl_2_ and 5% MgCl_2_; Volatile results are expressed as area units per gram of sample (AU × 10^4^/g of sample). Sig.: significance; ns: not significant; * *p* < 0.05; ** *p* < 0.01; *** *p* < 0.001; ^a–c^ Mean values in the same row (corresponding to the same parameter) followed by a different letter differ significantly (*p* < 0.05; Duncan’s test). *m*/*z*: Quantifier ion.

**Table 7:** Effect of NaCl replacement by other chloride salts on aldehyde, furans, acids, phenol and benzene-derived compounds, others and total volatile compounds content of dry-cured deer ‘‘cecina’’.

Volatile (AU × 10^4^/g of Cecina)	*m*/*z*	LRI	Control	Salt Mixture I	Salt Mixture II	SEM	Sig.
2-methylpropanal	72	548	12.81 ^a,b^	15.30 ^a^	11.68 ^b^	0.55	*
3-methylbutanal	58	656	98.89	118.39	100.64	5.49	ns
2-methylbutanal	58	669	67.11	83.82	72.19	3.91	ns
Hexanal	56	879	93.84 ^a^	59.43 ^b^	82.62 ^a^	4.71	**
Furan-2-carbaldehyde	96	947	25.13 ^a^	17.97 ^b^	30.13 ^a^	1.59	**
Benzaldehyde	105	1072	110.03	134.32	108.87	9.61	ns
2-phenylacetaldehyde	91	1152	231.46 ^a^	234.81 ^a^	146.51 ^b^	11.30	***
Nonanal	82	1184	10.72	8.77	8.71	0.58	ns
5-ethylfuran-2-carbaldehyde	124	1232	5.61	5.59	5.52	0.36	ns
Hexadecanal	82	1581	4.18	3.42	4.13	0.29	ns
Total aldehydes			659.77	681.81	571.02	22.62	ns
3-methylfuran	82	571	1.47	1.61	1.42	0.0880	ns
2-ethylfuran	81	703	6.87	6.03	5.20	0.38	ns
2,3,5-trimethylfuran	110	872	1.51 ^b^	2.71 ^a^	0.99 ^b^	0.20	***
2-butylfuran	81	963	4.65	3.49	3.60	0.28	ns
2-pentylfuran	81	1065	106.93 ^a^	66.65 ^b^	49.03 ^b^	6.14	***
2-ethenylfuran	94	1146	95.37	87.32	83.27	3.68	ns
1- (5-methylfuran-2-yl) ethanone	109	1147	20.97	21.14	19.28	1.08	ns
3-phenylfuran	144	1287	2.90 ^b^	2.86 ^b^	4.15 ^a^	0.25	*
Total furans			240.67 ^a^	191.81 ^b^	166.94^b^	9.42	**
Acetic acid	60	686	1261.07	1183.54	1143.65	51.66	ns
Propanoic acid	74	835	111.20	95.39	95.79	5.09	ns
2-methylpropanoic acid	73	903	153.48 ^a^	159.91 ^a^	79.96 ^b^	8.88	***
Butanoic acid	60	936	261.55	213.27	242.13	10.51	ns
3-methylbutanoic acid	60	994	1156.22 ^a^	1320.02 ^a^	474.30 ^b^	66.19	***
2-methylbutanoic acid	74	1001	417.61 ^a^	425.84 ^a^	197.65 ^b^	23.26	***
Pentanoic acid	60	1029	16.30	13.89	15.50	0.70	ns
2,2-dimethylpropanoyl 2,2-dimethylpropanoate	85	1062	7.47 ^a^	6.23 ^a,b^	5.39 ^b^	0.29	*
Hexanoic acid	60	1113	67.77	51.03	58.06	3.26	ns
Total acids			3452.67 ^a^	3469.13 ^a^	2312.42 ^b^	109.30	***
Toluene	92	812	43.41	54.41	56.22	2.52	ns
1,4-xylene	106	944	13.25	14.63	10.80	0.72	ns
2-methoxyphenol	109	1192	573.22	546.17	511.95	23.60	ns
4-methoxy-3-methylphenol	138	1257	26.14	25.56	21.78	1.57	ns
2,4-dimethylphenol	107	1262	9.48	9.71	8.36	0.48	ns
2-methoxy-5-methylphenol	138	1275	230.34	238.75	210.35	11.25	ns
1,3-dimethoxy-5-methylbenzene	152	1318	6.48	7.63	5.99	0.40	ns
4-ethyl-2-methoxyphenol	137	1338	76.28	75.63	59.34	4.51	ns
2,6-dimethoxyphenol	154	1398	31.65	32.18	26.50	1.33	ns
1,2,3-trimethoxy-5-methylbenzene	182	1414	2.61	3.48	2.85	0.15	ns
2-methoxy-4-prop-1-enylphenol	164	1456	2.20 ^b^	3.02 ^a^	1.56 ^b^	0.180	**
Phenol and benzene-derived compounds			1015.06	1011.20	915.71	40.34	ns
(E)-but-2-enedinitrile	78	641	2.92	2.57	3.03	0.18	ns
Mthylimino(oxo)methane	57	732	79.87	77.24	69.76	3.58	ns
Pyrazine	80	784	11.76 ^b^	15.16 ^a,b^	19.10 ^a^	0.98	**
Pyridine	79	804	10.98	10.51	13.82	0.72	ns
2,3-dihydro-1,4-dioxine	86	853	21.91	19.86	23.32	0.83	ns
Aniline	93	894	3.13	2.99	3.32	0.15	ns
Imidazolidine-2,4-dione	100	899	3.36	3.30	3.40	0.19	ns
2,6-dimethylpyrazine	108	1001	53.37 ^b^	91.97 ^a^	62.30 ^b^	3.79	***
Diethylcanamide	98	1005	9.71	8.63	7.29	0.53	ns
2,3-dimethylpyrazine	108	1010	31.86 ^b^	52.34 ^a^	19.27 ^c^	2.57	***
2,3,5-trimethylpyrazine	122	1088	213.23 ^b^	376.29 ^a^	179.47 ^b^	16.63	***
1H-imidazole-5-carbaldehyde	96	1196	4.61	4.82	4.46	0.25	ns
Total others			446.71 ^b^	665.69 ^a^	408.47 ^b^	21.08	***
Total compounds			12022.17 ^a^	12336.51 ^a^	10074.59 ^b^	224.01	***

Control: 100% NaCl; Salt mixture I: 30% NaCl and 70% KCl; Salt mixture II: 30% NaCl, 50% KCl, 15% CaCl_2_ and 5% MgCl_2_; Sig.: significance; ns: not significant; * *p* < 0.05; ** *p* < 0.01; *** *p* < 0.001; ^a–c^ Mean values in the same row (corresponding to the same parameter) followed by a different letter differ significantly (*p* < 0.05; Duncan’s test). *m*/*z*: Quantifier ion.
